# Complete mitochondrial genome of an extinct *Equus (Sussemionus) ovodovi* specimen from Denisova cave (Altai, Russia)

**DOI:** 10.1080/23802359.2017.1285209

**Published:** 2017-02-06

**Authors:** Anna S. Druzhkova, Alexey I. Makunin, Nadezhda V. Vorobieva, Sergey K. Vasiliev, Nikolai D. Ovodov, Mikhail V. Shunkov, Vladimir A. Trifonov, Alexander S. Graphodatsky

**Affiliations:** a Institute of Molecular and Cellular Biology SB RAS, Novosibirsk, Russia;; b Department of Natural Sciences, Novosibirsk State University, Novosibirsk, Russia;; c Institute of Archaeology and Ethnography SB RAS, Novosibirsk, Russia

**Keywords:** Equus, ovodovi, *Sussemionus*, equids, ancient, DNA, mitochondrial, genome

## Abstract

*Sussemionus* is an extinct subgenus of *Equus* first characterized and delineated in 2010. The almost complete mitochondrial genome is available only for a single specimen of *Sussemionus* – a 40,000 years old *E. ovodovi* from Proskuryakova cave (Khakassia, Russia). Our studies of ancient horses from Denisova cave (Altai, Russia) revealed mitochondrial DNA of this species in a 32,000 years old sample. Using alignments to multiple mitochondrial genomes of non-caballine equids, we recovered 100% complete mitochondrial genome of *E. ovodovi* for the first time. Phylogenetic analysis demonstrates close relationship between this individual and the one previously described in Khakassia.

The novel subgenus *Sussemionus* was delineated in the genus *Equus* by specific dental traits. The name *Sussemionus* was assigned due to osteological similarity to fossil horses from Sussenborn (Germany). The subgenus includes several species widely distributed from North America to Eurasia and Africa during Pleistocene (Eisenmann & Vasiliev 2011). Although plenty of fossil materials are available, only a single specimen was studied using molecular genetics techniques – *E. ovodovi* from Proskuryakova cave (Khakassia, Southwestern Siberia). The first efforts to sequence HVR-1 mtDNA fragment of three bone samples demonstrated that *E. ovodovi* belongs to non-caballine equids (Orlando et al. 2009). Sequencing of a complete mitochondrial genome from a single individual revealed that phylogenetically this species indeed does not belong to caballine equids, but rather occupies a basal position among all non-caballine equids (and is not closely related to any of living equid species) (Vilstrup et al. 2013). The sample was dated as 40,000 years before present with radiocarbon method, while it was earlier proposed that all representatives of subgenus *Sussemionus* were limited by Early and Middle Pleistocene (Eisenmann & Vasiliev 2011).

In this study we reconstructed the complete sequence of the mitochondrial genome of another individual of *Equus ovodovi* from Denisova cave (Altai, Southwestern Siberia), which is located 470 km southwest from the locality of the previous specimen. The DNA sequence was submitted to GenBank with accession number KY114520.

Sample preparation was performed at the Center of Cenozoic Geochronology Institute of Archaeology & Ethnography Russian Academy of Sciences, Siberian Branch (Novosibirsk), radiocarbon dating performed in CAIS (Center for Applied Isotope Studies, University of Georgia, USA) (Labcode 00741) indicated the sample age as 32,360 ± 450 years. Ancient DNA was extracted as described in Druzhkova et al. (2013). Prior to sequencing, libraries were enriched using hybridization with contemporary *E. caballus* biotinilated mtDNA immobilized on Dynabeads^®^ Streptavidin magnetic beads (Life Technologies, USA) as in Maricic et al. (2010). Paired-end sequencing was performed on Illumina MiSeq using Reagent Kits v2, 500-cycles.

A total of 8,506,508 read pairs were obtained. PALEOMIX BAM Pipeline v1.2.5 (Schubert et al. 2014) was used for sequencing data processing. 3,235,963 (38.0%) of read pairs were successfully trimmed and collapsed (AdapterRemoval v. 2.1.7). Due to phylogenetical proximity of *E. ovodovi* to modern non-caballine equids (Vilstrup et al. 2013), we used complete mitochondrial genomes of *E. asinus*, *E. hemionus* and *E. kiang* as alternative references in addition to horse (*E. caballus*) used in previous study (GenBank: NC_001788.1, NC_016061.1, NC_020433.1, and NC_001640.1, respectively). Alignment was performed with bowtie2 v. 2.2.9 with ‘–very-sensitive-local’ option, and minimum mapping quality 30. Alignment refinement included PCR duplicate removal, quality rescaling (mapDamage v. 2.0.6), and indel realignment (GATK v. 3.3-0-g37228af). As a result, from 15,084 (*E. caballus* mtDNA as reference) to 17,590 (*E. hemionus*) aligned collapsed read pairs remained. Consensus sequences were compared and corrected manually (Geneious v. 8.1.6) resulting in complete (100%) recovery of the mitochondrial genome. The genome obtained in our study does not possess the cluster of tandem repeats in the control region (such cluster is present in *E. caballus* and many other mammalian species). Previous *E. ovodovi* mitogenome assembly (Vilstrup et al. 2013) was considered incomplete because authors assumed the presence of such cluster. Here, we demonstrate the absence of any tandem repeats due to significant coverage of overlapping reads in this region.

To determine the phylogenetic position of the sample, we used all sequences from the study reconstructing the relationships among extinct and extant equids (Vilstrup et al. 2013). Alignment was performed with MAFFT v.7.245. Partitioning and model selection for BI were performed in PartitionFinder v.1.1.1. Optimal partitioning scheme included 5 partitions: RNA genes + intergenic spacers (model GTR + G + I), protein-coding genes codon 1 (GTR + G + I), 2 (HKY + G + I), and 3 (GTR + G), and control region (HKY + G). The phylogenetic tree was constructed in MrBayes v.3.2.5 using partitioning and models as above, chain length 50,000,000, sample frequency 1000, first 25% trees discarded as burn-in. Alternative ML tree was constructed with RaxML v.8.2.3, partitioning as above, model GTR + G for all partitions. The resulting tree with BI consensus topology is presented in Figure 1. Both ML and BI analyses yielded our sample position close to *E. ovodovi* from Khakassia. 118 nucleotide changes separate two *E. ovodovi* specimens compared to 45–163 (mean 109) in contemporary *E. caballus*, which indicates that both *E. ovodovi* specimens belong to the same species.

**Figure 1.**
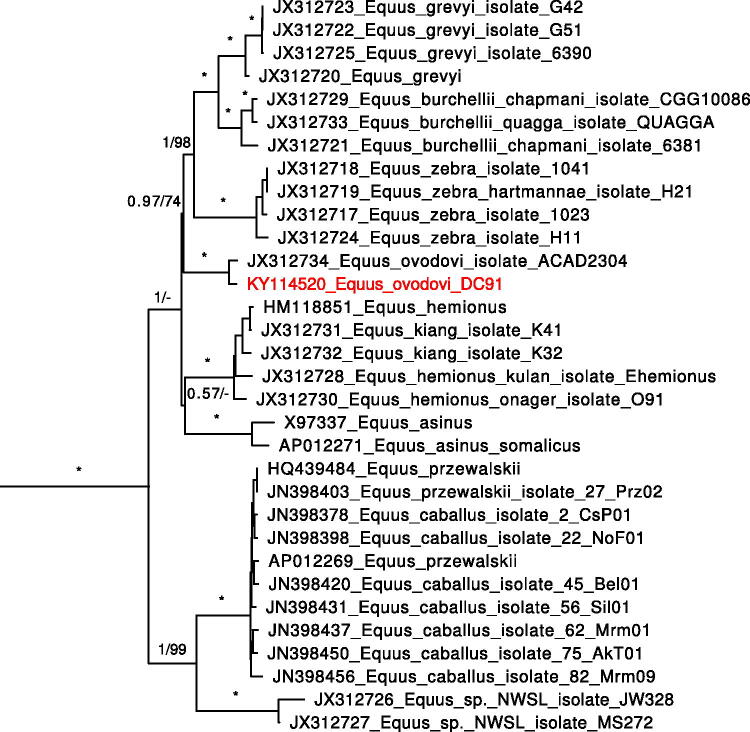
Phylogenetic tree based on complete mitochondrial genomes including equid samples used in Vilstrup et al. (2013) together with *Equus ovodovi* sample from Denisova cave. Support values: posterior probabilities from 4250 of 5000 trees sampled by MrBayes/bootstrap support from 1000 replicates by RaxML. ‘*’ indicates complete support by both methods (1/100), ‘-’ indicates differing branching pattern for RaxML tree.

Our data confirmed the results obtained based on the sample from Khakassia (Eisenmann & Vasiliev 2011; Vilstrup et al. 2013) and extend the geographic range and time of existence of *E. ovodovi* species. Our data point that *E. ovodovi* is closer to zebras than to *E. asinus*, *E. hemionus* or *E. kiang*, while previous study (Vilstrup et al. 2013) indicates the basal position of *E. ovodovi* in all non-caballine equids. This incongruence might have arose due to the difference in alignment partitioning between the studies. Either way, both studies provide a low support in the resolution of the polytomy of these four groups (*E. ovodovi*, *E. asinus*, *E. hemionus* +* E. kiang*, and zebras).

Many unresolved phylogenetic issues of equids were considerably improved by the application of nuclear markers (Orlando 2014). We expect that scrutiny investigation of these markers in ancient *E. ovodovi* samples will shed light on the exact phylogenetic position of this species among non-caballine equids.
